# Susceptibility loci in lung cancer and COPD: association of IREB2 and FAM13A with pulmonary diseases

**DOI:** 10.1038/srep13502

**Published:** 2015-08-27

**Authors:** Iwona Ziółkowska-Suchanek, Maria Mosor, Piotr Gabryel, Marcin Grabicki, Magdalena Żurawek, Marta Fichna, Ewa Strauss, Halina Batura-Gabryel, Wojciech Dyszkiewicz, Jerzy Nowak

**Affiliations:** 1 Institute of Human Genetics, Polish Academy of Sciences, Strzeszyńska 32, Poznań, Poland; 2 Department of Thoracic Surgery, University of Medical Sciences, 62 Szamarzewskiego Street, 60-569 Poznań, Poland; 3 Department of Pulmonology, Allergology and Respiratory Oncology, Poznań University of Medical Sciences, 84 Szamarzewskiego Street, 60-569 Poland; 4 Department of Endocrinology, Metabolism and Internal Diseases, Poznań University of Medical Sciences, 49 Przybyszewskiego Street, Poland; 5 Laboratory for Basic Research and Translational Medicine in Vascular Diseases, Clinic of Internal and Vascular Surgery, Poznan University of Medical Sciences, Dluga ½ Street, 61-848 Poland.

## Abstract

Genome-wide association studies have identified loci at 15q25 (*IREB2*) and 4q22 (*FAM13A*), associated with lung cancer (LC) and chronic obstructive pulmonary disease (COPD). The aim of our research was to determine the association of *IREB2* and *FAM13A* SNPs with LC and severe/very severe COPD patients. We examined *IREB2* variants (rs2568494, rs2656069, rs10851906, rs13180) and *FAM13A* (rs1903003, rs7671167, rs2869967) among 1.141 participants (468 LC, 149 COPD, 524 smoking controls). The frequency of the minor *IREB2* rs2568494 AA genotype, was higher in LC *vs* controls (*P* = 0.0081, OR = 1.682). The *FAM13A* rs2869967 was associated with COPD (minor CC genotype: *P* = 0.0007, OR = 2.414). The rs1903003, rs7671167 *FAM13A* variants confer a protective effect on COPD (both *P* < 0.002, OR < 0.405). Haplotype-based tests identified an association of the *IREB2* AAAT haplotype with LC (*P* = 0.0021, OR = 1.513) and *FAM13A* TTC with COPD (*P* = 0.0013, OR = 1.822). Cumulative genetic risk score analyses (CGRS), derived by adding risk alleles, revealed that the risk for COPD increased with the growing number of the *FAM13A* risk alleles. OR (95% CI) for carriers of ≥5 risk alleles reached 2.998 (1.8 to 4.97) compared to the controls. This study confirms that the *IREB2* variants contribute to an increased risk of LC, whereas *FAM13A* predisposes to increased susceptibility to COPD.

Chronic obstructive pulmonary disease (COPD) and lung cancer (LC) are caused by the interaction between genetic susceptibility and environmental influences. Both diseases are leading causes of mortality and morbidity worldwide. In Poland, COPD is responsible for approximately 15.000 patients’ deaths per year and the clinical features of COPD are observed in about 10% of the Polish population, over 40 years of age^1^. Patients with COPD appear to be at increased risk of developing lung cancer, suggesting that there is a common mechanism which induces both diseases^2^. In addition, it was reported that the presence of mild emphysema, even without demonstrable obstruction, independent of cigarette-smoking burden, confers a substantial risk of lung cancer^2^. Although cigarette smoking is the main environmental risk factor, only about 10–20% of smokers develop COPD^2^ and only 10 to 15% of lung cancer cases arise in individuals who have never smoked^3^. Familial aggregation of both diseases has been observed. The connection between family history of LC and COPD was reported in a segregation analysis, in relatives of non-smoking cases^4^. Twin studies have also demonstrated that pulmonary function is heritable^5^. These data suggest a common underlying genetic determinant of susceptibility to lung diseases.

Several genome-wide association studies (GWASs) have identified genomic regions associated with COPD, LC and impaired lung function. These GWAS *loci* are located at *IREB2* (iron-responsive element binding protein 2, MIM 613299) gene on chromosome 15q24-25.1^6^,^7^,^8^,^9^ and in the 4q22.1 *locus*, including *FAM13A* (family with sequence similarity 13, member A, MIM 147582) gene^10^,^11^,^12^.

The *IREB2* gene region (15q24) contains a number of genes encoding nicotinic acetylcholine receptor subunits (*CHRNA3/CHRNA5/CHRNAB4* cluster for instance), which show strong association with COPD. This region was equally associated with LC^6^ and nicotine addiction^13^. The genes *CHRNA3/CHRNA5* has been associated with the risk for nicotine dependence through mRNA brain expression levels^14^, dense genotyping^15^ and GWAs^16^ in various populations, including smokers with normal lung function and lung diseases^17^, like lung cancer and COPD. The identification of 15q24 region as a susceptibility loci in lung diseases, may suggest that LC and COPD are closely related and should not be considered separately, with particular emphasis on overlapping genetic effects^18^. Additionally, with the use of candidate gene approach, loci on 4q24 (*INTS12*), 6p21 (*AGER*) and 5p15 (*ADCY2*) have demonstrated the association with COPD^19^. An alternative approach to assess the cumulative effect of GWAs susceptibility in lung cancer after sub-phenotyping of COPD were submitted by Young R. *et al.* They have demonstrated that the risk genotypes from previously reported GWAs loci (including *FAM13A* and *CHRNA3/5*), incorporated into the algorithm with clinical variables, may estimate an overall genetic risk score^20^.

The *IREB2* gene belongs to a group of genes which regulate mammalian iron homeostasis. IREB2 registers cytosolic iron status mainly through an iron-sulfur switch mechanism^21^. Considering its role as mediator of iron homeostastis and observed phenotype of *IREB2* knock-out models, *IREB2* gene is a candidate that could have an impact on many diseases’ pathogenesis. Coon *et al.* (2006) examined *IREB2* gene polymorphisms in Alzheimer’s disease patients and demonstrated that two single nucleotide polymorphisms (SNPs) (rs2656070 and rs13180) were significantly associated with this disorder^22^. DeMeo *et al.* (2009) identified genomic regions from 56 lung tissue gene expression microarrays and used them to select SNPs, of which three *IREB2* variants were associated with Norwegian COPD patients. In addition, the same investigators have observed increased levels of IREB2 mRNA and protein in lung tissue samples from COPD cases compared to healthy controls, which allowed them to propose *IREB2* as a novel COPD susceptibility gene^7^.

The biological function of the *FAM13A* gene product is poorly understood. The highest expression of *FAM13A* gene was detected in the brain and ovaries, followed by the lungs and kidneys^23^. This gene encodes a protein with two coiled-coil domains and three nuclear localization signals^24^. It is suggested that the most important part of the FAM13A protein is its N-terminal extension containing the Rho-GAP domain, which presents tumour suppressor activity through inhibition of the intracellular signal transduction molecule RhoA^18^. It was shown that genetic variants in *FAM13A* gene may determine susceptibility to COPD and lung cancer^18^. The Rho GTPases activity regulation may indicated the potential role of FAM13A in carcinogenesis. Several such modulators of these type in immune cell migration and inflammation have been developed for cancers^25^. Recently, Z. Jin, J. Chung *et al.* have shown that knockdown of FAM13A significantly reduces the Wnt signaling activity in A549 human lung cancer cells^26^. Rho GTPases are also involved in the pulmonary endothelial barrier function in the lungs^27^, which is often dysregulated in lung diseases, such COPD and asthma^28^.

The current study aimed to determine the association of previously reported *IREB2* and *FAM13A* SNPs with lung cancer and chronic obstructive pulmonary disease among two selected groups of Polish patients and smoking controls. The results of this study may elucidate whether genetic predisposition to COPD is shared with that of LC, and suggest a new pathway in lung disease development.

## Results

### Genotype and allele distribution among patients and controls

The genotype/allele frequencies are summarized in Tables 1, 2, 3, [Table t2], [Table t2], [Table t2], [Table t3]. The observed genotype frequencies of these 7 polymorphisms were all in agreement with the Hardy-Weinberg equilibrium (HWE) in the control subjects (for *IREB2* HWE, the p*-*values were as follows: 0.674, 0.167, 0.149, 0.794; for *FAM13A* HWE p-values were: 0.986, 0.844, 0.766). We found that the *IREB2* variants demonstrated an association with LC cases, while the *FAM13A* SNPs were associated with COPD.

For the *IREB2* rs2568494 polymorphism, the AA genotype frequency, compared with the proportion of the GG genotype, was significantly higher in LC cases (*P* = 0.0081) than in the controls. Similarly, the presence of GA+AA genotypes (*P* = 0.0129) and the A allele (*P* = 0.0043) were more common among LC patients as compared to controls in single polymorphism analyses (Table 1). No association of *IREB2* rs2568494 genotypes with the results of the pulmonary function tests: FEV1 and FEV1/VC (FEV1- forced expiratory volume in 1 second; VC- vital capacity; FEV1/VC- ratio) and smoking exposure in LC cases (Table 4) was noticed. We observed that frequencies of the G allele and GG genotype at rs2568494, G allele and GG genotype at rs10851906 and CC genotypes at rs13180 SNPs, were higher in controls compared to LC cases (Tables 1 and 3). The occurrence of the GG genotype of rs10851906 was differed greatly between LC cases and controls (*P* = 0.0027). When the *IREB2* association was examined in the lung cancer cases without COPD (N = 369) compared to controls the results were comparable to those received for general lung cancer group (N = 468). In case of rs2568494 SNP the association was found but was not significant after Bonferroni correction (AA genotype: *P* = 0.0206, OR = 1.62 [1.08–2.44] and GA+GG: *P* = 0.0457, OR = 1.32 [1–1.73]; A allele: *P* = 0.0154, OR = 1.27 [1.05–1.55]). The occurrence of rs10851906 GG genotype was different between LC no-COPD cases and controls (*P* = 0.0087, OR = 0.4 [0.2–0.81]). The frequencies of CC genotype and C allele at rs13180, were higher in controls compared to LC no-COPD cases (CC genotype: *P* = 0.0098, OR = 0.57 [0.37–0.88]; CT+CC genotype: *P* = 0.015, OR = 0.71 [0.55–0.94] and C allele: *P* = 0.0052, OR = 0.75 [0.62–0.92]).

**Figure 1:**
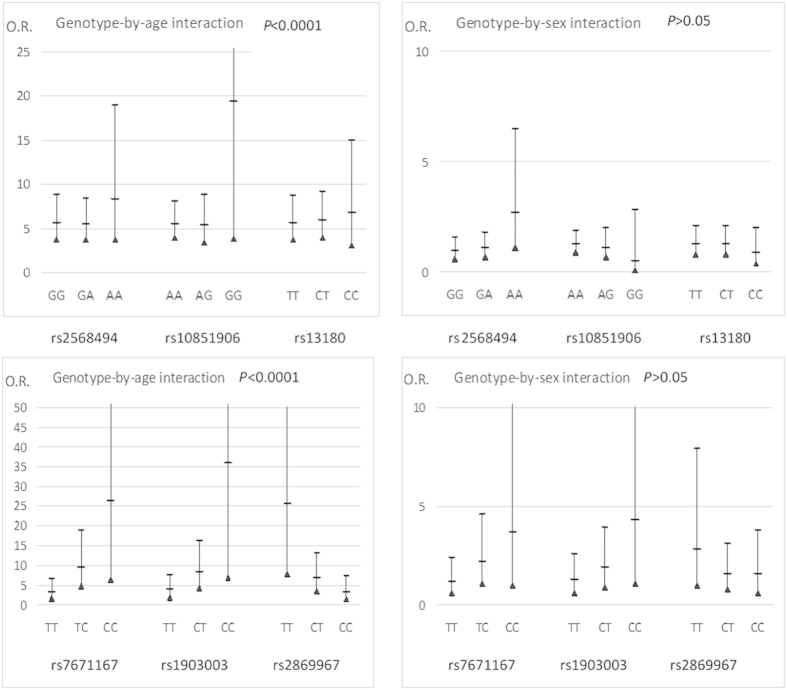
Significant influences of *IREB2* and *FAM13A SNPs* on COPD and lung cancer according to age and sex. A multiple linear regression analysis was performed for *IREB2* (rs2568494, rs10851906, rs13180) genotypes in LC and *FAM13A* (rs7671167, rs1903003, rs2869967) genotypes in COPD. The odds ratios with 95% confidence intervals (O.R. with 95%CI) for genotypes of selected SNPs are displayed. Control group was set as reference group. All results for age variable were statistically significant. LC: lung cancer; COPD: chronic obstructive pulmonary disease.

The interactions of selected genotypes of *IREB2* (rs2568494, rs10851906, rs13180) with age that influenced the LC risk were observed (Fig. 1). For three *IREB2* SNPs the results obtained for genotypes interacted with age were: rs2568494 GG OR = 5.7 95%CI [3.7–8.9], GA OR = 5.6 95%CI [3.7–8.4], AA OR = 8.3 95%CI [3.7–18]; rs10851906 AA OR = 5.6 95%CI [3.0–8.1], AG OR = 5.5 95%CI [3.4–8.9], GG OR = 19.3 95%CI [3.9–97] and rs13180 TT OR = 5.7 95%CI [3.7–8.8], CT OR = 6 95%CI [4–9.2], CC OR = 6.8 95%CI [3.1–15]. No significant associations were found in case of sex variable (*P* > 0.05) (Fig. 1).

Each of the three *FAM13A* SNPs showed a significant difference in genotype/allele frequency between COPD cases and controls (Tables 2, 3). The rs2869967 CC genotype was closely associated with COPD (*P* = 0.0007), also when the COPD with LC+COPD patients was analyzed (combined COPD with LC+COPD; *P* < 0.0001). Similarly, the frequency of the C allele was significantly higher in these two groups (*P* = 0.0009 and 0.0001, respectively) compared with the controls. We found no association of *FAM13A* rs2869967 genotypes with lung function measurements FEV1 and FEV1/FVC (FVC- forced vital capacity; FEV1/FVC - ratio) and smoking exposure in the COPD (Table 4). The TC, CC and TC+CC genotypes of rs7671167 SNP were more common in the control group as compared to COPD (*P* = 0.022, < 0.0001, 0.0015) and COPD with LC+COPD groups (*P* = 0.036, 0.0004, 0.003) (Table 2). These results were consistent with the difference in C allele frequency between these two groups of patients (*P* = 0.0002, 0.0003) (Table 3). Likewise, in case of rs1903003 SNP, the presence of the CT, CC and CT+CC genotypes was significantly more frequent among the controls compared to COPD (*P* = 0.024, 0.0014, 0.0028) and COPD with LC+COPD groups (*P* = 0.028, 0.0002, 0.0019) (Table 2). These observations were compatible with C allele distribution in these two analyzed groups of COPD patients analyzed (*P* = 0.0009, 0.0002) (Table 3). The interactions of *FAM13A* (rs1903003, rs7671167, rs2869967) genotypes with age that influenced the COPD risk were detected (Fig. 1). For each *FAM13* SNPs the results obtained for genotypes interacted with age were: rs7671167 TT OR = 3.3 95%CI [1.6–6.6], TC OR = 9.5 95%CI [14.8–18], CC OR = 26 95%CI [6.5–105]; rs1903003 TT OR = 4 95%CI [2–7.7], CT OR = 8.4 95%CI [8.4–16], CC OR = 36 95%CI [7–136] and rs2869967 TT OR = 25 95%CI [8–81.6], CT OR = 7 95%CI [3.6–13], CC OR = 3.3 95%CI [1.4–7.5]. No significant associations were found in the case of the sex variable (*P* > 0.05). None of the *FAM13A* SNPs showed significant associations with lung cancer cases (Fig. 1).

### Multiple testing of SNP-SNP interactions

Logistic regression was used to adjust the effects of studied SNPs in impact in occurrence of LC (4 SNPs in *IREB2* gene), COPD, and combined COPD with LC+COPD (3 SNPs in *FAM13A* gene). Each analysis including all studied SNPs from particular gene as covariate. In LC, *IREB2* rs252568494 revealed the strongest association (*P* = 0.037; OR = 1.2; 95%CI: 1–1.5) after correction of influence of the three other SNPs. In COPD, *FAM13A* rs7671167 was the most significantly associated (*P* = 0.029; OR = 0.7; 95%CI: 0.5–0.97), whereas in combined COPD with LC+COPD, the *FAM13A* rs2869967 was the most relevant (*P* = 0.04; OR = 1.3; 95%CI: 1–1.7).

### Linkage disequilibrium analysis

**Figure 2:**
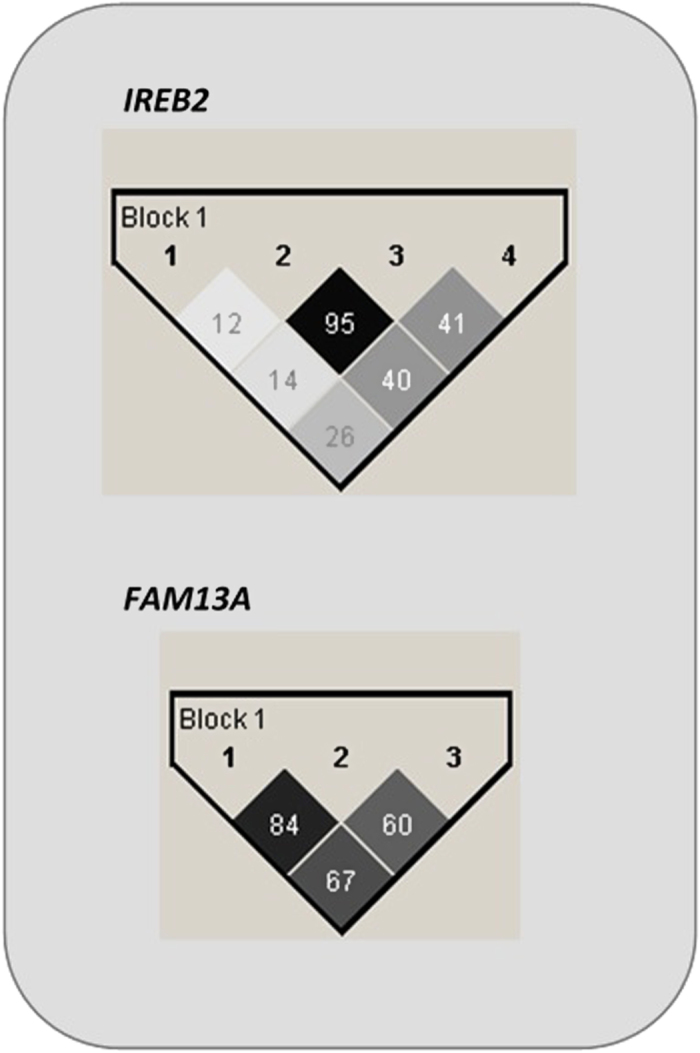
Pairwise linkage disequilibrium between SNPs in *IREB2* gene and *FAM13A* gene.

Linkage disequilibrium (LD) calculations (Levontin’s *D’* value and correlation coefficient r^2^) were done for each pair of the 4 investigated *IREB2* polymorphisms and 3 SNPs of *FAM13A* gene. The values for LD between SNPs are shown in Fig. 2. The pairwise LD values suggested strong linkage between studied SNPs in both genes. Within *IREB2* gene there is strong LD between rs2656069 and rs10851906 (r^2^ = 0.95). Within *FAM13A,* SNPs rs7671167 and rs1903003 are in LD (r^2^ = 0.84).

### Frequency of *IREB2* and *FAM13A* haplotypes

To analyze the combined effect of these four investigated *IREB2* SNPs and 3 SNPs of *FAM13*, we the generated the haplotypes on the basis of the genotyping data observed. The construction of haplotypes revealed the presence of seven *IREB2* haplotypes and 6 *FAM13A* haplotypes in COPD/LC patients and controls. Haplotypes with a frequency lower than 1% in all groups were not considered for further analysis. Finally, we analyzed the eight most common haplotypes (four for each gene; Table 5). The most frequent haplotypes in patients and controls were: AAAT (36%), GAAT (27%), GGGC (21%), GAAC (14%) for *IREB2* and CCT (43%), TTC (41%), TTT (10%), CTT (3%) for *FAM13A*. The *IREB2* AAAT haplotype was associated with an increased risk of LC (*P* = 0.0021; OR = 1.51; 95%CI: 1.62–1.97). In the case of *FAM13A* gene, the frequencies of haplotype TTC were significantly higher in COPD patients and COPD combined with LC+COPD group than in the controls (*P* = 0.0013; OR = 1.82; 95%CI: 1.26–2.63; and *P* = 0.0003; OR = 1.76; 95%CI: 1.29–2.39, respectively).

### Cumulative genetic risk score

**Figure 3:**
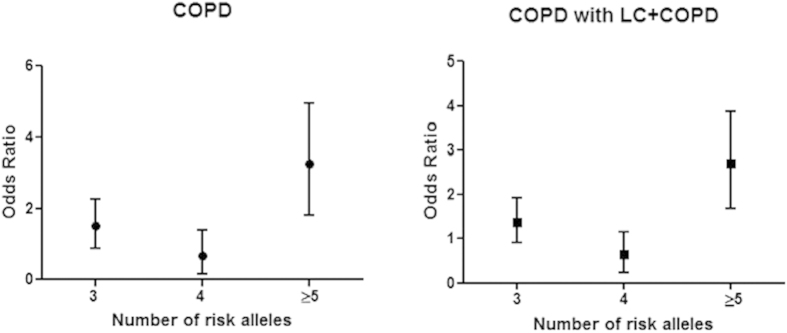
Cumulative genetic risk score (CGRS) analysis of *FAM13A* SNPs. The effect of unweighted cumulative genetic risk score on COPD and COPD with LC+COPD was calculated using logistic regression analysis. The odds ratios (black symbols) with 95% confidence intervals (range bars) for the number of risk alleles at each of *FAM13A* SNPs are represented from unweigheted analysis. COPD: chronic obstructive pulmonary disease; LC: lung cancer; COPD with LC+COPD: COPD with 40% admixture of subjects with concomitant LC.

All study variants in both genes indicated a significant association with LC or COPD were included in the cumulative genetic risk score analysis (CGRS). The CGRS of two *IREB2* SNPs (rs2568494, rs13180) among LC did not a reveal significant increase in OR value: for carriers of ≥3 risk alleles OR reached 1.5957 (1.1717 to 2.1730). In *FAM13A* gene the risk alleles were defined as T for rs13180, rs7671167 and A for rs2568494. The average (±SD) of cumulative risk scores among COPD patients (3.54 ± 1.97) and mixed COPD with LC+COPD group (3.459 ± 1.96) were significantly higher than the controls (2.857 ± 1.83) with a *P* value < 0.0001. We also observed a significant difference in the average (±SD) of weighted cumulative risk scores comparing COPD and COPD with LC+COPD to the control group (3.54 ± 1.98 and 3.46 ± 1.96 *vs*. 2.86 ± 1.83, respectively, *P* < 0.0001). Individuals were stratified according to the number of risk alleles into group carrying ≤2 (reference group), 3, 4 and ≥5 alleles. The risk of COPD increased with the growing number of the *FAM13A* risk alleles. OR (95% CI) for COPD carriers of ≥5 risk alleles reached 2.998 (1.8092 to 4.9678) for unweighted CGRS (Fig. 3). In COPD with LC+COPD group, odds ratio for carriers of ≥5 risk alleles amounted to 2.542 (1.6705 to 3.8693) for unweighted CGRS (Fig. 3). The odds ratios calculated in unweighted and weighted CGRS analysis were similar, which is probably connected with the low number of risk alleles and small sample size.

### Statistical power analysis

The post-hoc analysis revealed that the statistical power of our study for analyses of the differences in genotype distribution between subgroups of patients and controls ranged between 80–97%. The statistical power of the study to detect the *IREB2* alleles associated with LC (OR: 0.7 to 1.4) for the three SNPs with a frequency of 0.2 to 0.38 (in the additive model) was 80–97%. The analysis of rs10851906 alleles in LC group, in the recessive model, revealed that the power of study is 80%. In case of *FAM13A* alleles associated with COPD and combined COPD with LC+COPD group (OR: 0.5 to 1.7) with a frequency of 0.3 to 0.5 (in the additive model) the statistical power was 91–97%. This means that the study had sufficient power to detect an association of the *IREB2* gene in LC and *FAM13A* gene in COPD and combined COPD with LC+COPD group, in the case-control analysis.

## Discussion

In this research we examined 7 SNPs among patients with LC, COPD and in healthy controls from Poland (n = 1141), in an attempt to evaluate the association of *IREB2* and *FAM13A* loci with these diseases. In our study we observed that *IREB2* variants appeared to be associated with LC, whereas the *FAM13A* was linked with COPD.

The *IREB2* variants have been previously linked to COPD as well as lung cancer. Three independent groups performing GWA studies have identified 15q25 locus, comprising the *IREB2* gene, as associated with lung cancer^6^,^8^,^9^. In our study, three out of four selected SNPs showed a significant association with the LC, whereas none of them reached the level of significance among COPD cases (Table 2). The genotype AA of *IREB2* rs2568494 was significantly more frequent among LC group (*P* = 0.0081; OR = 1.682), which suggested that this variant might increase the risk of lung cancer. This association was independent of lung function among cases (FEV1 and FEV1/VC) and smoking intensity (Table 6). However, the effect of the abovementioned variant and age of lung cancer development was observed (Fig. 1). The prevalence of rs10851906 GG and rs13180 CC genotypes was significantly higher among the controls compared with LC cases, which suggests that minor alleles of these SNPs may have an apparently protective effect (OR = 0.379, 0.595, respectively). Our results are difficult to interpret because of the low number of reports on the protective effect of single *IREB2* gene polymorphisms. Among 4 *IREB2* haplotypes, only one was associated with an increased risk of LC. In the current study, the *IREB2* locus was also examined in the lung cancer cases without COPD and the results just reflect the overall case-control relationship. However, it is worth noting that the lung cancer patients selected for this study, were only those considered for surgery. This may explain the low rate of COPD patients in this group (21%), whereas the prevalence of COPD in several lung cancer cases is reported to between 50–70%^18^,^29^. An alternative approach to estimate an overall genetic risk score, by the multivariate analysis that include genotypes and clinical variables which are independent predictors of lung cancer, were proposed by R. Young *et al.* In this report, the SNPs data were used to compile the risk model, which includes many risk genotypes from genes implicated in both COPD and lung cancer. These multifactorial approach may be helpful in identifying genes underlying the development of lung cancer^20^.

In contrast to COPD, the potential role of the *IREB2* gene in human cancer is not well documented. Recently, studies on mouse models showed that overexpression of *IREB2* promoted the growth of tumor xenografts in nude mice. In addition, the authors have noticed that the unique conserved insert of 73 amino acids of IREB2 mammalian protein, contributes to IREB2 pro-oncogenic potential and is essential for accelerated tumor growth^30^. However, a quantitative analysis of *IREB2* mRNA levels in paired normal lung and lung *adenocarcinoma* tissue demonstrated the increased expression of *CHRNA5*, but no change in *IREB2*^31^. In another study, using gene overexpression and/or gene knockdown and apoptosis analyses, the authors have failed to reveal that *IREB2* mediates effects on lung cancer cell growth *in vitro*^32^. Further studies are needed to prove the link between IREB2 protein and cancer biology.

DeMeo *et al.* (2009) have demonstrated that *IREB2* may be a COPD susceptibility gene, identified through the integration of lung microarray expression data and replicated genetic association in many Caucasian cohorts^7^,^33^,^34^, including the Polish population^35^. Nevertheless, we were not able to confirm these findings in our group of Polish patients with COPD. *IREB2* is located in the region near the *CHRNA3/CHRNA5/CHRNAB4* genes cluster with a possible role of lung diseases development. The significant LD in this region a is source of difficulties in association analyses. M. Hardin *et al.* (2012) have demonstrated a significant association of *IREB2* rs13180 with 315 severe COPD cases from Poland (*P* = 3.4 × 10^−3^; OR = 0.69)^35^. However, after adjustment for the presence of the *CHRNA3/5* SNP, this association was no longer significant, indicating that the positive results may be driven by LD with *CHRNA3/5* polymorphism.

The functional consequences of *IREB2* polymorphism are unclear. The protein product of the *IREB2* gene is involved in maintaining human cellular iron metabolism. It has been demonstrated that smokers had higher concentrations of iron in the lungs^36^. Iron imbalance may lead to oxidative injury, whereas an excess of iron has an impact upon regional inflammation in the lungs, which may be relevant to the pathogenesis of COPD and lung cancer. It is supposed that *IREB2* variants may further affect COPD and lung cancer when coupled with the increased levels of iron accumulated through exposure to cigarette smoke^7^.

We confirmed previous observations, which suggest that *FAM13A* variants may play a role in COPD susceptibility for all three SNPs selected for this study. The statistically significant values were obtained in almost each genotype with a minor allele in COPD patients and in the COPD group merged with patients suffering from concomitant LC and COPD (Table 3). It is not surprising, that the results for all SNPs were similar, due to the high level of linkage disequilibrium between these three SNP (CEU HapMap, NCBI B36 assembly)^18^. Among three *FAM13A* variants, only rs2869967 showed an association with an increased risk of COPD, whereas rs7671167 and rs1903003 were significantly more frequent among the controls (Table 3). A positive effect of these SNPs on age at COPD occurrence was observed (Fig. 1).

The rs2869967 CC genotype was significantly more frequent among the COPD group (*P* = 0.0007; OR = 2.414) and in the COPD group combined with LC+COPD (*P* < 0.0001; OR = 2.358). This variant has been mainly investigated in large Chinese populations and its CC genotype frequency seemed to be increased^37^,^38^. Wang *et al.* showed that rs7671167 and rs2869967 were associated with the FEV1/FVC ratio among all subjects, whereas rs2869967 revealed only a borderline association (*P* = 0.05) with COPD cases after adjusting for age, gender, body mass index (BMI), pack-years of smoking and current smoking status^38^. Further investigations including functional analyses are warranted to confirm the impact of CC rs2869967 variant in the modulation of the COPD risk.

The prevalence of CC genotypes of rs7671167 and rs1903003 was significantly elevated among the controls compared with COPD cases, which suggests the protective effect of these alleles (Table 3). Here, we confirm the findings of M. Cho *et al.* among non-Hispanic white, African-American and Norwegian cases, followed by R. Young *et al.* in Caucasians. In the first study, conducted among 2,940 COPD cases and 1,380 controls with normal lung function, the locus most highly associated with COPD included two SNPs: rs1903003 and rs7671167 in high linkage disequilibrium (*r*^*2*^ = 0.85). Subsequently, they genotyped these two SNPs in 502 COPD cases and 504 controls and in two large family-based cohorts including 3,808 cases and confirmed the associations. Similarly, the study by R. Young *et al.* demonstrated that the C allele of rs7671167 is associated with a reduced risk of COPD^18^. In addition, they have indicated its protective effect in COPD combined with lung cancer patients with pre-existing COPD, which is in agreement with our observations in COPD combined with LC+COPD. However, these authors also showed, that this variant was associated with reduced risk of lung cancer (OR = 0.75, *P* = 0.002). This relationship was absent in our study, because no significant difference was detected in the frequencies of genotypes/alleles of *FAM13A* among lung cancer patients. Another study^35^, carried out among 305 Polish patients with severe and very severe COPD, did not demonstrate a significant association between the rs7671167 SNP and COPD. However, they indicated that the C allele was associated with improved lung function, both in COPD cases and control population^35^. The association of *FAM13A* variants with lung function was replicated in several studies^11^,^12^. D. Hancock *et al.* meta-analyzed GWA studies, including 20.890 participants^11^. A phenotype, described by two important lung-function parameters: FEV1 and FEV1/FVC, is inheritable and provides the basis for evaluation of lung function. Hancock *et al.* identified eight loci associated with FEV1/FVC, including also variants in the *FAM13A* gene, namely rs2869967, which was analysed in our study. In current study, we did not identify an association between this SNP and FEV1 and FEV1/FVC parameters, as well as smoking intensity (Table 6). Among the 4 *FAM13A* haplotypes selected for this study, the prevalence of TTC was significantly higher in COPD patients than in controls.

We also observed a cumulative effect of three SNPs in the *FAM13A* locus. The risk of COPD escalated with an increasing number of risk alleles. Although the tendency for increasing OR for individuals with four risk alleles was moderate, probably due to the small number of carriers in this group, among carriers of ≥5 risk alleles OR rose significantly to 2.998. These results might suggest an additive effect of the variants being studied.

Whether any of these three SNPs is functional remains unknown, although given their location, an effect on splice variants would be possible. The most statistically significant result in our study was obtained for rs7671167 SNP, which lies in intron 4 of the *FAM13A* gene and was identified by Cho *et al.* It localizes downstream of the most important part of the FAM13A molecule: the Rho-GAP domain. Although little is known of the FAM13A function, the Rho GTPase activating role suggests both anti-inflammatory and tumor suppressor activity for FAM13A protein. *FAM13A* gene expression analyses in cell lines from several tissues (not comprising the lung) have shown a consistent increase in its expression in response to hypoxia^39^. Differences in the expression of this gene have also been noted in respiratory epithelial cells during differentiation into pulmonary type II alveolar cells *in vitro* model and among individuals with mild as compared to severe cystic fibrosis^40^. Recently, Z. Jin, J. Chung *et al.* showed that depletion of FAM13A in human lung cancer cells causes a reduction in Wnt signaling activity which provides evidence that Fam13a may contribute to human lung diseases^26^.

The effects of studied SNPs in impact in LC occurrence, COPD, and combined COPD with LC+COPD were analysed using multiple testing of SNP-SNP interactions. In LC, *IREB2* rs252568494 revealed the strongest association. In COPD, *FAM13A* rs7671167 was the most significantly associated, whereas in combined COPD with LC+COPD, the *FAM13A* rs2869967 was the most relevant. After adjustment of the influence of the other SNPs in each gene, these associations were still significant (*P* < 0.05). Despite the loss of significance after Bonferroni correction, the direction of SNP effect remained the same. It is possible that the strong LD between certain SNP may cause the decrease of *P*-value level. These SNPs may be independent predictors of phenotype in pulmonary diseases and should be considered in future studies concerning the development of gene-based prognostic scores for LC and COPD.

There are some limitations to the current study that need to be addressed. The number of COPD patients is limited, but the group was very accurately selected and the history of the disease and also exposition to noxious substances were collected in detail. 75% of cases had severe or very severe airflow limitation in the course of the disease (stage III and IV in GOLD classification), which represents more extreme phenotype of this disease. Although limited by our sample size, we will still able to demonstrate several significant associations in case of both genes in all groups of cases *vs* controls. With a Bonferroni correction for multiple testing, which is very conservative approach, the associations between *IREB2* and lung cancer and *FAM13A* and COPD remain significant. Although, many data had provided evidence for a role of *IREB2* gene in COPD, we were not able to demonstrated this associations. It is possible that association in *IREB2* locus may be driven by LD with *CHRNA3/5* SNPs only, but these variants were not examined in current study. It is also possible that *IREB2* SNPs have not an impact in severe/very severe COPD occurrence. We did not demonstrated significant associations, however small effect was found in rs2568494 AA genotype in the combined COPD with LC+COPD group. Although the cohort of COPD cases were pooled restrictively, it seems to be underpowered to detect an effect at the *IREB2* locus. However, the statistical power for our analyses ranged between 80–97%, which may suggest that the study had sufficient power to detect an association.

Another limitations of the current study, is connected with smoking controls selection. Controls were matched by sex with both groups of patients (~70% of males in all groups). However, in the case of age distribution considerable deviation was observed between COPD and lung cancer cases and controls (median age 66, 64 and 55 years, respectively), which may lead to bias being introduced. The smoking status of controls was not matched exactly, because information about smoking exposure measures (like cigarettes/day or pack-years) was not available for 40% of controls. In this subgroup, we were not able to calculate pack years, because no information about all smoking exposure measures were pooled.

The control group included only cancer-free cases with normal pulmonary function, which allowed us to simultaneously compare COPD and lung cancer groups with the matched controls. This study design enabled us to determine the possible link between COPD and lung cancer patients related to *IREB2* and *FAM13A* variants. To our knowledge, this is the first report in which *IREB2* and *FAM13A* genes were analyzed in parallel among COPD and lung cancer cases, with cumulative genetic risk score analyses. This approach provided an assessment of the potential role of *IREB2* and *FAM13A* genes in lung disease development in the Polish population. To date, only R. Young *et al.* have examined one variant of the *FAM13A* gene in case-control studies of COPD, lung cancer and controls^11^.

## Conclusion

We confirmed the association between *IREB2* gene and lung cancer and between *FAM13A* gene and COPD in Polish patients. Further studies are required to elucidate the functional role of these variants, which may have an impact on lung disease susceptibility.

## Methods

All experiments were performed in accordance with relevant guidelines and regulations approved by the Ethics Committee of the Poznan University of Medical Sciences (decision no. 802/10). All subjects gave their written informed consent to participate.

### Materials

All subjects were of Caucasian ancestry, recruited from Wielkopolska region in Poland. The detailed characteristics of patients and controls are displayed in Table 6.

### LC Patients

The cohort of 468 LC cases was recruited in the Wielkopolska Center of Pulmonology and Thoracosurgery from patients scheduled for surgical treatment. Most of them were males (69%), with median age of 63.5 years (range 51–78 years). The diagnosis of lung cancer was confirmed by the histopathological examination of the resected or biopsied tissue specimens in all cases. 97% of cases were non-small cell lung cancer (NSCLC). Spirometry was done in all LC patients and the median values of measurements were as follows: FEV1%: 89%, VC%: 99% and FEV1/VC: 94%. 91% patients had positive history of tobacco smoking.

### COPD Patients

COPD subjects (N = 149, 72% males) were recruited in the Department of Pulmonology, Allergology and Respiratory Oncology, Poznan University of Medical Sciences. Median age of COPD patients was 66 years (range 39–87 years; of which 40 cases = <60 years). Inclusion criteria for stable COPD comprised airflow limitation as indicated by post-bronchodilator FEV_1_/FVC <0,7 and FEV_1_ ≤70% of normal predicted values. Patients with established diagnosis of asthma, LC, history of atopy, known AAT (α_1_-antitrypsin) deficiency or serum AAT level of less than 1.0 g/L were excluded from the study. Severity of airflow limitation was classified according to GOLD standard (Global Initiative for Chronic Obstructive Lung Disease) as follows: II—moderate, III—severe, IV—very severe. Most of them had positive history of tobacco smoking (97.7%). The mean value of pack-years of smoking was 43 ± 6.9.

### COPD with LC+COPD Patients

In 99 (21%) out of 468 LC patients, COPD was diagnosed as the concomitant disease, which allowed us to distinguish the subgroup of LC patients with coexisting COPD (LC+COPD, N = 248). This subgroup was combined with COPD patients and analyzed separately as the COPD subjects with 40% admixture of subjects with concomitant LC (COPD with LC+COPD).

### Controls

Control subjects (N = 524) demonstrated normal lung function with no evidence of airflow limitation (FEV_1_/FVC > 0.7). This group consisted of individuals for screening check-up in hospital or healthy blood donors with negative history of medical illnesses recruited at the Regional Blood Transfusion Centre. Efforts were undertaken to frequency-match cases by ethnicity, age and sex. Most of them were males (72%), with median age of 55 years (range 35–80 years). All controls were treated as smokers: current (29%), former (71%). Among former smokers were two groups: quitting smoking less than year (23%) and more than year (48%).

### Methods

DNA samples from COPD patients, LC patients and controls were isolated from peripheral blood lymphocytes by Gentra Puregene Blood Kit (Qiagen, Hilden, Germany). DNA purity and concentration was confirmed using NanoDrop ND-1000 spectrophotometer.

We selected the SNPs previously associated with lung function and/or COPD in GWAs. We chose genomic regions based on review of the literature, and used the most significant reported SNPs which were analyzed in relatively large groups of cases. For *IREB2* gene we selected the SNPs described by D. De Meo *et al.*^7^, for *FAM13A* gene we chose the SNPs which were identified by M. Cho *et al.*^10^. All polymorphisms selected for this study had minor allele frequencies >0.4 in order to achieve sufficient statistical power. Altogether four SNPs in *IREB2* (rs2568494, rs2656069, rs10851906, rs13180) and three variants of *FAM13A* (rs1903003, rs7671167, rs2869967) were analysed.

The SNPs were genotyped using pre-designed TaqMan® SNP genotyping assays (Life Technologies, Carlsbad, California; assays IDs: *IREB2*: 16043098_10, 15916464_10, 11522538_10, 8873396_1; *FAM13A*: 1143659_10, 1143656_10, 15837681_10)^41^. The PCR was performed with HOT FIREPol Probe qPCR Mix Plus (no ROX) according to the manufacturer’s instructions provided by Solis Biodyne (Tartu, Estonia). The PCR thermal cycling was as follows: initial denaturation at 95 °C for 15 min.; 40 cycles of 95 °C for 15 sec and 60 °C for 60 sec. Thermal cycling was performed using a CFX96 Touch™ Real-Time PCR Detection System (Bio-Rad, Hercules, California, U.S.). As a quality control measure, negative controls and approximately 5% of samples were genotyped in duplicate to check genotyping accuracy. The genotypes of selected samples were confirmed by direct sequencing (OLIGO, IBB, Warszawa, Poland).

### Statistical analysis

The Hardy–Weinberg equilibrium (p^2^ + 2pq + q^2^ = 1, where p is the frequency of the variant allele and q = 1−p) was tested by chi-square test to compare the observed and expected genotype frequencies in healthy controls. The statistical differences in genotype and allele frequencies between the studied groups and controls were also evaluated by the chi-square test. For all polymorphisms, the ancestral or major allele was considered wild-type and the homozygous wild-type genotype was considered the referent genotype. Odds ratios (ORs) and 95% confidence intervals (CIs) for association between the *IREB2* or *FAM13A* genotypes and alleles and studied diseases were estimated using logistic regression analysis. Each patients group (COPD and LC) was examined as an independent case series. An extended COPD cohort, including COPD cases and LC patients with coexisting COPD (COPD with LC+COPD) was additionally considered in a separate case-control analysis. Statistical calculations were performed using GraphPad PRISM 5, module Statistical Analysis, Contingency Tables (GraphPad Software Inc.San Diego, CA).

Linkage disequilibrium (LD) measures were used to detect associations between the 4 investigated *IREB2* polymorphisms and 3 SNPs of *FAM13A* gene. On the basis of the genotype data of all subjects, pair-wise LD was calculated using 2 standardized LD coefficients: r^2^ and Levontin’s *D’*. These associations were also tested for significance by means of the chi-square test. The expectation-maximization (EM) algorithm was applied to generate maximum likelihood estimates of haplotype frequencies. It was assumed that the Hardy–Weinberg equilibrium is applicable to the constructed haplotypes based on observed genotypes of *IREB2* and *FAM13A* genes. Finally, the associations between specific haplotypes and the disease were studied among patients and controls (using chi-square test). To account for false-positive findings, multiple testing Bonferroni correction was applied. Because we tested 2 genes in 2 groups of patients, the result is significant if the corrected P-value is below the cutoff of <0.0125 [P-value * n (number of genes in test) <0.05]^42^. Calculations were performed using HAPLOVIEW, Linkage format [http://www.broad.mit.edu/mpg/haplo-view]. In case of two SNPs, which appeared significantly more frequent in patients’ groups in relation to controls, frequency of genotypes, lung function measures and smoking exposure were compared by One-way ANOVA, Ordinary test. Association between rs2568494 *IREB2* variant and lung function measures (FEV1, FEV1/VC) was analyzed in the lung cancer cases, whereas rs2869967 *FAM13A* variant and COPD phenotype (FEV1, FEV1/FVC) was compared among COPD patients. Smoking exposure was set as a quantitative feature (pack-years of smoking). A multiple linear regression analysis was used to investigate the correlation of variables, including age, sex, and genotype with disease by *Logistic Regression 05.07.20* program (http://statpages.org/logistic.html). Effects of selected *IREB2* and *FAM13A* genotypes on COPD/LC development, according to sex and age were estimated with the use of control group as a reference category. Logistic regression was used to multiple testing of SNP-SNP interactions by STATISTICA version 10.0 software. QUANTO software (version 1.2.4) was employed for sample size calculations.

### Cumulative genetic risk score

SNPs in *FAM13A* showing significant association with COPD were included in the cumulative genetic risk score (CGRS) analysis. Genotypes for each SNP were coded as 0, 1 or 2 indicating the number of COPD risk alleles in the genotype. Unweighted and weighted CGRS (wCGRS) were calculated. In an unweighted approach the number of risk variants carried by an individual at each *FAM13A* SNP was counted to create cumulative genetic risk score (possible score for three SNPs range 0–6). In a weighted approach, OR estimated for risk allele in current study was used (T *vs*. C, OR 1.634; 1.565 and C *vs*. T, OR 1.548 of rs7671167, rs1903003 and rs2869967, respectively). A weighted risk score is the sum of the weighted COPD allele counts, weighted by natural logarithm of odds ratio for each risk allele ln(OR) and scaled by factor 3/w_1_ + w_2_ + w_3_ where w_i_ = ln(OR) for the i^th^ SNP and i = 1 to 3^43^. Only samples without missing genotype were included in the analysis (COPD n = 149, Controls n = 524).

The effect of unweighted and weighted cumulative genetic risk score on COPD was calculated using logistic regression analysis. To compare the average (±SD) of CGRS and wCGRS between COPD patients and controls the unpaired t test was applied.

## Additional Information

**How to cite this article**: Ziółkowska-Suchanek, I. *et al.* Susceptibility loci in lung cancer and COPD: association of IREB2 and FAM13A with pulmonary diseases. *Sci. Rep.* **5**, 13502; doi: 10.1038/srep13502 (2015).

## Figures and Tables

**Table 1:** Logistic regression analysis for the associations of the *IREB2* SNPs with COPD and LC.

SNP	Frequency of genotypes		OR (95% CI)	*P*	Frequency of genotypes	OR (95% CI)	*P*	Frequency of genotypes	OR (95% CI)	*P*
	Controls	COPD			LC			COPD with LC+COPD		
rs2568494										
GG	0.44	0.43	1^1^		0.36	1^1^		0.39	1^1^	
GA	0.44	0.44	1.04 [0.70–1.53]	0.859	0.47	1.30 [0.99–1.70]	0.058	0.46	1.19 [0.86 –1.65]	0.294
AA	0.12	0.13	1.09 [0.61–1.95]	0.775	0.17	**1.68 [1.14–2.48]**	**0.0081**^**2**^	0.15	1.36 [0.85–2.19]	0.201
GA+AA	0.56	0.57	1.05 [0.73–1.51]	0.806	0.64	1.38 [1.07–1.78]	0.0129	0.61	1.23 [0.90 –1.67]	0.192
rs2656069										
AA	0.59	0.64	1^1^		0.63	1^1^		0.63	1^1^	
AG	0.34	0.32	0.88 [0.59–1.30]	0.523	0.33	0.89 [0.68 –1.17]	0.415	0.33	0.93 [0.67–1.28]	0.645
GG	0.07	0.04	0.54 [0.22–1.33]	0.176	0.04	0.58 [0.33–1.03]	0.060	0.04	0.50 [0.23–1.06]	0.065
AG+GG	0.41	0.36	0.82 [0.56–1.20]	0.312	0.37	0.84 [0.65 –1.09]	0.187	0.37	0.85 [0.63–1.17]	0.321
rs10851906										
AA	0.59	0.64	1^1^		0.63	1^1^		0.63	1^1^	
AG	0.34	0.32	0.87 [0.59–1.29]	0.487	0.34	0.94 [0.72–1.23]	0.671	0.33	0.94 [0.68–1.29]	0.684
GG	0.07	0.05	0.64 [0.27–1.48]	0.289	0.03	**0.38 [0.20–0.73]**	**0.0027**^**2**^	0.04	0.50 [0.23–1.06]	0.066
AG+GG	0.41	0.36	0.83 [0.57–1.21]	0.332	0.37	0.85 [0.66 –1.10]	0.209	0.37	0.86 [0.63–1.18]	0.346
rs13180										
TT	0.37	0.40	1^1^		0.45	1^1^		0.41	1^1^	
CT	0.47	0.45	0.88 [0.59–1.30]	0.515	0.44	0.79 [0.60–1.03]	0.084	0.45	0.89 [0.64–1.24]	0.495
CC	0.16	0.15	0.92 [0.53–1.58]	0.753	0.11	**0.60 [0.40–0.89]**	**0.0102**^**2**^	0.14	0.81 [0.50–1.28]	0.361
CT+CC	0.63	0.60	0.89 [0.61–1.29]	0.523	0.55	0.74 [0.57–0.95]	0.0209	0.59	0.87 [0.64–1.19]	0.376

COPD—chronic obstructive pulmonary disease; LC—lung cancer; COPD with LC+COPD: the associations with COPD were studied twice: using pure COPD group and COPD with 40% admixture of subjects with concomitant LC.

^1^Reference category; OR (95% CI), odds ratio (95% confidence interval).

^2^**Result statistically significant** (P < 0.0125, after Bonferroni correction).

**Table 2 t2:** Logistic regression analysis for the associations of the *FAM13A* SNPs with COPD and LC.

SNP	Frequency of genotypes		OR (95% CI)	*P*	Frequency of genotypes	OR (95% CI)	*P*	Frequency of genotypes	OR (95% CI)	*P*
	Controls	COPD			LC			COPD with LC+COPD		
rs7671167										
TT	0.24	0.38	1^1^		0.28	1^1^		0.35	1^1^	
TC	0.50	0.47	0.62 [0.41–0.93]	0.0223	0.44	0.79 [0.58–1.06]	0.118	0.49	0.69 [0.49–0.98]	0.036
CC	0.26	0.15	**0.19 [0.09 –0.37]**	**<0.0001**^2^	0.28	0.95 [0.67–1.34]	0.761	0.16	**0.45 [0.29–0.70]**	**0.0004**^**2**^
TC+CC	0.76	0.62	**0.54 [0.36–0.79]**	**0.0015**^**2**^	0.72	0.84 [0.63–1.12]	0.230	0.65	**0.61 [0.44–0.85]**	**0.003**^**2**^
rs1903003										
TT	0.27	0.40	1^1^		0.32	1^1^		0.38	1^1^	
CT	0.51	0.47	0.63 [0.42–0.94]	0.024	0.46	0.77 [0.58 –1.04]	0.084	0.49	**0.69 [0.49–0.96]**	0.028
CC	0.22	0.13	**0.41 [0.23–0.71]**	**0.0014**^**2**^	0.22	0.85 [0.60–1.20]	0.355	0.13	**0.42 [0.26–0.67]**	**0.0002**^2^
CT+CC	0.73	0.60	**0.56 [0.38–0.52]**	**0.0028**^**2**^	0.68	0.80 [0.61 –1.05]	0.103	0.62	**0.60 [0.44–0.83]**	**0.0019**^**2**^
rs2869967										
TT	0.34	0.23	1^1^		0.35	1^1^		0.23	1^1^	
CT	0.49	0.49	1.41 [0.90–2.20]	0.134	0.43	0.86 [0.65–1.14]	0.295	0.49	1.44 [0.48–1.00]	0.052
CC	0.17	0.28	**2.41 [1.44–4.05]**	**0.0007**^**2**^	0.22	1.27 [0.89–1.81]	0.193	0.28	**2.36 [1.53–3.64]**	**<0.0001**^**2**^
CT+CC	0.66	0.77	**1.66 [1.09–2.53]**	**0.0170**^**2**^	0.65	0.96 [0.74–1.25]	0.782	0.77	**1.67 [1.18–2.36]**	**0.0034**^**2**^

COPD—chronic obstructive pulmonary disease; LC—lung cancer; COPD with LC+COPD: the associations with COPD were studied twice: using pure COPD group and COPD with 40% admixture of subjects with concomitant LC.

^1^Reference category; OR (95% CI), odds ratio (95% confidence interval).

^2^**Result statistically significant** (P < 0.0125, after Bonferroni correction).

**Table 3 t3:** The allele frequency distribution and logistic regression analysis of the *IREB2* and *FAM13A* SNPs in COPD and LC.

SNP	Frequency of genotypes		OR (95% CI)	*P*	Frequency of genotypes	OR (95% CI)	*P*	Frequency of genotypes	OR (95% CI)	*P*
Allele	Controls	COPD			LC			COPD with LC+COPD		
rs2568494										
G	0.66	0.65	1^1^		0.60	1^1^		0.62	1^1^	
A	0.34	0.35	1.04 [0.80–1.26]	0.765	0.40	**1.31 [1.09–1.57]**	**0.0043**^2^	0.38	1.18 [0.94–1.50]	0.152
rs2656069										
A	0.76	0.68	1^1^		0.79	1^1^		0.80	1^1^	
G	0.24	0.32	0.81 [0.59–1.11]	0.178	0.21	0.82 [0.67–1.02]	0.074	0.20	0.2 [0.63–1.06]	0.126
rs10851906										
A	0.76	0.80	1^1^		0.80	1^1^		0.80	1^1^	
G	0.24	0.20	0.83 [0.60–1.13]	0.234	0.20	0.79 [0.64–0.98]	0.032	0.20	0.82 [0.63–1.07]	0.137
rs13180										
T	0.61	0.62	1^1^		0.67	1^1^		0.64	1^1^	
C	0.39	0.38	0.94 [0.72–1.22]	0.631	0.33	**0.77 [0.64–0.94]**	**0.0064**^**2**^	0.36	0.89 [0.72 –1.12]	0.321
rs7671167										
T	0.49	0.61	1^1^		0.50	1^1^		0.59	1^1^	
C	0.51	0.39	**0.61 [0.47–0.79]**	**0.0002**^2^	0.50	0.97 [0.82–1.16]	0.767	0.41	**0.68 [0.54–0.84]**	**0.0003**^**2**^
rs1903003										
T	0.52	0.63	1^1^		0.55	1^1^		0.62	1^1^	
C	0.48	0.37	**0.64 [0.49–0.83]**	**0.0009**^**2**^	0.45	0.91 [0.76–1.08]	0.285	0.38	**0.66 [0.53–0.82]**	**0.0002**^2^
rs2869967										
T	0.58	0.48	1^1^		0.56	1^1^		0.48	1^1^	
C	0.42	0.52	**1.55 [1.20–2.00]**	**0.0009**^**2**^	0.44	1.10 [0.91–1.30]	0.349	0.52	**1.53 [1.23–1.89]**	**0.0001**^**2**^

COPD—chronic obstructive pulmonary disease; LC—lung cancer; COPD with LC+COPD: the associations with COPD were studied twice: using COPD group and COPD with 40% admixture of subjects with concomitant LC.

^1^Reference category; OR (95% CI), odds ratio (95% confidence interval).

^2^**Result statistically significant** (P < 0.0125, after Bonferroni correction).

**Table 4:** Association between *IREB2* rs2568494 and *FAM13A* rs2869967 SNPs with lung function measures and smoking exposure in the COPD and LC.

		Genotype	Lung function measures		Pack-years of smoking
			FEV1 [% pred.]	FEV1/VC [%]	
LC	*IREB2*	GG	84	92	44.2
	rs2568494	GA	82	92	46.6
		AA	85	91	43.5
		***P***	0.364	0.94	0.408
			FEV1 [% pred.]	FEV1/FVC [%]	
COPD	*FAM13A*	TT	43	56	47.3
	rs2869967	CT	40.7	52.3	39.8
		CC	41.4	53.7	45.1
		***P***	0.816	0.335	0.454

LC—lung cancer; COPD—chronic obstructive pulmonary disease; FEV1—forced expiratory volume in 1 second; VC—vital capacity; FVC—forced vital capacity; FEV1/VC, FEV1/FVC—ratio; % pred.—% of predicted value; ***P***(One-way ANOVA—Ordinary test).

**Table 5:** Frequency distribution of the *IREB2* and *FAM13A* haplotypes and results of logistic regression analysis for association with COPD, LC, COPD+LC with COPD cases and controls.

SNP	Frequency of genotypes		OR (95% CI)	*P*	Frequency of genotypes	OR (95% CI)	*P*	Frequency of genotypes	OR (95% CI)	*P*
	Controls	COPD			LC			COPD with LC+COPD		
IREB2										
AAAT	0.34	0.31	0.87 [0.59–1.29]	0.479	0.39	**1.51 [1.16–1.97]**	**0.0021**^1^	0.35	1.05 [0.76–1.44]	0.761
GAAT	0.27	0.28	1.06 [0.70–1.58]	0.793	0.27	0.91 [0.75–1.31]	0.950	0.26	0.97 [0.68–1.65]	0.795
GGGC	0.24	0.16	0.61 [0.38–0.98]	0.039	0.20	0.79 [0.59–1.07]	0.134	0.18	0.70 [0.48–1.02]	0.065
GAAC	0.15	0.17	1.14 [0.69–1.86]	0.612	0.13	0.84 [0.59–1.21]	0.356	0.16	1.80 [0.72–1.64]	0.705
FAM13A										
CCT	0.43	0.36	0.76 [0.52–1.1]	0.143	0.45	1.09 [0.85–1.4]	0.497	0.37	0.78 [0.57–1.70]	0.123
TTC	0.37	0.52	**1.82 [1.26–2.63]**	**0.0013**^**1**^	0.42	1.27 [0.96–1.6]	0.103	0.51	**1.76 [1.29–2.39]**	**0.0003**^**1**^
TTT	0.12	0.10	0.83 [0.45–1.49]	0.511	0.08	0.63 [0.41–0.96]	0.032	0.08	0.64 [0.38–1.09]	0.097
CTT	0.03	0.02	0.65 [0.19–2.23]	0.499	0.04	1.34 [0.68–2.65]	0.391	0.03	0.92 [0.37–2.27]	0.860

COPD—chronic obstructive pulmonary disease; LC—lung cancer; COPD with LC+COPD: the associations with COPD were studied twice: using pure COPD group and COPD with 40% admixture of subjects with concomitant LC.

OR (95% CI), odds ratio (95% confidence interval).

^1^**Result statistically significant** (P < 0.0125, after Bonferroni correction).

**Table 6:** Characteristics of patients with lung cancer (LC, n = 468), chronic obstructive pulmonary disease (COPD, n = 149) and controls (n = 524).

Variable		LC		COPD	Controls
		n (%)		n (%)	n (%)
Age	≤60	148 (32)		40 (27)	374 (70)
	>60	320 (68)		109 (73)	150 (30)
Gender	Male	325 (69)		107 (72)	379 (72)
	Female	143 (31)		42 (28)	145 (28)
Smoking status	Yes	442 (94)		146 (98)	all
	No	22 (5)		3 (2)	—
	Passive	4 (1)		3 (2)	—
	Current	438 (94)		31 (21)	151 (29)
	Former	30 (6)		118 (79)	373 (71)
Pack-years of smoking	≤30	87 (19)		46 (31)	—
	>30	355 (76)		103 (69)	—
Family history	Yes	146 (31)		—	—
	No	245 (53)		—	—
	No information	77 (16)		—	—
Other	LC Histology		COPD Severity^1^		
	NSCLC	456 (97)	II	37 (25)	
	*Sq. c. carcinoma*	208 (46)	III	69 (46)	
	*Adenocarcinoma*	185 (41)	IV	43 (29)	
	*other*	62 (13)			
	SCLC	12 (3)			
	TNM Staging^2^				
	I	162 (36)			
	II	154 (34)			
	III	120 (26)			
	IV	18 (4)			

NSCLC—non-small cell lung cancer; SCLC—small cell lung cancer; “–”—no information.

^1^Severity of COPD was classified according to GOLD standard: II—moderate, III—severe, IV—very severe.

^2^Information available for 454 participants.
